# Ongoing Clinical Trials in Aging-Related Tissue Fibrosis and New Findings Related to AhR Pathways

**DOI:** 10.14336/AD.2021.1105

**Published:** 2022-06-01

**Authors:** Hang-Xing Yu, Zhe Feng, Wei Lin, Kang Yang, Rui-Qi Liu, Jia-Qi Li, Xin-Yue Liu, Ming Pei, Hong-Tao Yang

**Affiliations:** ^1^Department of Nephrology, First Teaching Hospital of Tianjin University of Traditional Chinese Medicine, Tianjin, China.; ^2^National Clinical Research Center for Chinese Medicine Acupuncture and Moxibustion, Tianjin, China.; ^3^Shaanxi University of Chinese Medicine, Xianyang, Shaanxi, China.; ^4^Kidney Disease Treatment Center, The first affiliated hospital of Henan university of CM, Zhengzhou, Henan, China

**Keywords:** fibrosis, tryptophan, metabolites, aryl hydrocarbon receptor, aging, gut microbiota

## Abstract

Fibrosis is a pathological manifestation of wound healing that replaces dead/damaged tissue with collagen-rich scar tissue to maintain homeostasis, and complications from fibrosis contribute to nearly half of all deaths in the industrialized world. Ageing is closely associated with a progressive decline in organ function, and the prevalence of tissue fibrosis dramatically increases with age. Despite the heavy clinical and economic burden of organ fibrosis as the population ages, to date, there is a paucity of therapeutic strategies that are specifically designed to slow fibrosis. Aryl hydrocarbon receptor (AhR) is an environment-sensing transcription factor that exacerbates aging phenotypes in different tissues that has been brought back into the spotlight again with economic development since AhR could interact with persistent organic pollutants derived from incomplete waste combustion. In addition, gut microbiota dysbiosis plays a pivotal role in the pathogenesis of numerous diseases, and microbiota-associated tryptophan metabolites are dedicated contributors to fibrogenesis by acting as AhR ligands. Therefore, a better understanding of the effects of tryptophan metabolites on fibrosis modulation through AhR may facilitate the exploitation of new therapeutic avenues for patients with organ fibrosis. In this review, we primarily focus on how tryptophan-derived metabolites are involved in renal fibrosis, idiopathic pulmonary fibrosis, hepatic fibrosis and cardiac fibrosis. Moreover, a series of ongoing clinical trials are highlighted.

## 1.Introduction

Fibrosis is a pathological extension of the wound healing process with high mortality that is majorly characterized by the formation and deposition of excess fibrous connective tissue resulting in progressive architectural remodeling in nearly all tissues and organs, eventual organ deformation and functional failure [1, 2]. It is difficult to diagnose at early stage and the pathogenesis is quite complex, so the efficacy and prognosis are not satisfactory, which is one of the major problems in clinical medical research. Aging is a main risk factor for numerous pathologies, and the risk of tissue fibrosis, including renal fibrosis, idiopathic pulmonary fibrosis (IPF), hepatic fibrosis and cardiac fibrosis, is significantly elevated with age [3]. In addition, progressive fibrosis is recognized as a sign of aging in many organs. Indeed, nearly half of deaths are attributed to the complications of organ fibrosis with the progression of industrialization, highlighting the importance of fibrosis inhibition in maintaining human health [4]. Fibrosis is often observed in many organs, causing architecture disruption and ultimately leading to irreversible damage to organ function, which includes but is not limited to the lung, kidney, liver and heart [5]. Although organ fibrosis was previously recognized as irreversible, recent advances indicate that certain circumstances permit fibrosis regression when the preventable causes are eradicated, bringing renewed hope that fibrosis could be curable instead of a death sentence [6].

Aryl hydrocarbon receptor (AhR) is a highly conserved environment-sensing transcription factor that integrates dietary, microbial, environmental, and metabolic cues to regulate complex transcriptional programs in a ligand-specific and cell-type specific manner [7-9]. It is composed of an amino (N-) terminal bHLH domain required for DNA binding, followed by two PER-ARNT-SIM (PAS) domains (A and B) and a carboxy (C-) terminal transactivation domain (TAD) [10]. In the cytoplasm, AhR forms a stable complex with HSP90 molecules, the AhR-interacting protein (HBV X associated protein 2), the co-chaperone p23 and the protein kinase SRC. Once activated by its ligand, the binding of a ligand at the PAS domain of the AhR leads to a conformational change in the exposing of the exposing of the nuclear localization signal. AhR dissociates from the complex and enters the nucleus. In the nucleus, AhR forms a dimer with the AhR nuclear translocator (ARNT) that regulates downstream gene transcription, and phosphorylation modification of AhR enhances its biological activity. Both AhR and ARNT can recruit a co-activator and then initiate the transcription of the AhR target genes, which including CYP1A1 and CYP1B1, the most topical genes and encode CYP1 family enzymes. Other AhR target genes include AhRR, INF-α [11] and Nrf2 [12]. AhR signaling also has crosstalk with other pathways such as Wnt/β-catenin, transforming growth factor-β/Smad (TGF-β/Smad) [13], nuclear factor-κB (NF-κB) [14], the renin-angiotensin system (RAS) [15] and matrix metalloproteinase (MMP) [16] due to the molecular interaction between activated AhR and other proteins, thereby regulating the cell functions (Fig. 2).

AhR ligands are considerably selective in their activity, and the pharmacokinetic processes of ligands may be very different [17]. Well-known high-affinity AhR ligands include various toxic and hydrophobic chemicals, such as polychlorinated biphenyls, polycyclic aromatic hydrocarbons, and halogenated compounds [18]. A key to understanding the biological function of AhR is to identify endogenous ligands with high affinity, especially tryptophan derivatives. However, compared to TCDD, the affinities of most tryptophan metabolites, including kynurenine [19], tryptamine, indole acetic acid [20], indole, 3-methylindole, indole-3-aldehyde, indole-3-lactic acid, indole-3-propionic acid, and indole-3-pyruvate are quite low [21]. The discovery of 6-formylindolo[3,2-b] carbazole (FICZ) as an endogenous and high affinity agonist of AhR fills this gap. FICZ has been proven to be more potent than TCDD and can be efficiently metabolized by cytochrome CYP1A1, CYP1A2 and CYP1B1, thus keeping its concentration at a low level [22]. Nevertheless, its level is elevated by other AhR antagonists due to the inhibition of CYP1A1, CYP1A2 and CYP1B1, and AhR is subsequently activated [22]. In addition to mediating the toxic effects of various environmental pollutants, such as carcinogenic, teratogenic, and inflammatory effects, the functional role of AhR also plays an important role in various physiopathological processes, such as immune regulation [23], fibrosis, growth and development [24], and maintenance of physiological homeostasis.

AhR is implicated in various aging-related diseases and tissue fibrosis, as it modulates numerous genes that are involved in many molecular pathways and cellular processes [25, 26]. For example, klotho is a well-known aging suppressor [27], the expression of which is significantly decreased, likely through an AhR-associated mechanism after treatment with TCDD [28]. Sirtuins (Sirt) are other prominent regulators of replicative lifespan and AhR can accelerate senescence by suppressing the activity of Sirtl [29] and Sirt3 [30]. Moreover, from the outcome of recent physiological and epidemiological studies, it appears that a considerable portion of the environmental influence on human disease is mediated by microbial communities, highlighting the importance of gut microbiota intervention in therapeutic strategy development [31]. Although the complex interaction between the host and resident gut microbiota remains elusive, tryptophan metabolism is recognized to play a crucial role in microbiota-host crosstalk through the AhR signaling pathway [32-34]. Furthermore, coronavirus disease 2019 (COVID-19) is a severe respiratory disease caused by respiratory syndrome coronavirus 2 (SARS-CoV-2), which severely harms human health. A large amount of epidemiological, viral immunological and current clinical evidence supported the possibility of pulmonary fibrosis as one of the major complications in patients with COVID-19. In severe cases of COVID-19 infection, pulmonary fibrosis is rapidly progress at patient’s imaging [35]. At present there are no reports on the mechanism of COVID-19 induced pulmonary fibrosis. However, with the existing theoretical basis we find that in addition to pulmonary fibrosis caused by the virus itself, other factors also play an extremely important role such as normal immune mechanism and abnormal immune mechanism, possibly as a consequence of a cytokine storm [36]. George et al. found that COVID-19 shared major risk factors with IPF, and anti-fibrotic therapies for IPF may also have value in preventing or alleviating severe COVID-19, fueling considerable enthusiasm for exploiting new drugs simultaneously targeting COVID-19 and fibrosis [37]. Considering that coronaviruses could activate the AhR signaling pathway after entry into cells [38], a promising treatment for both COVID-19 and tissue fibrosis may be achieved by targeting the tryptophan-AhR pathway.

**Figure 1.**
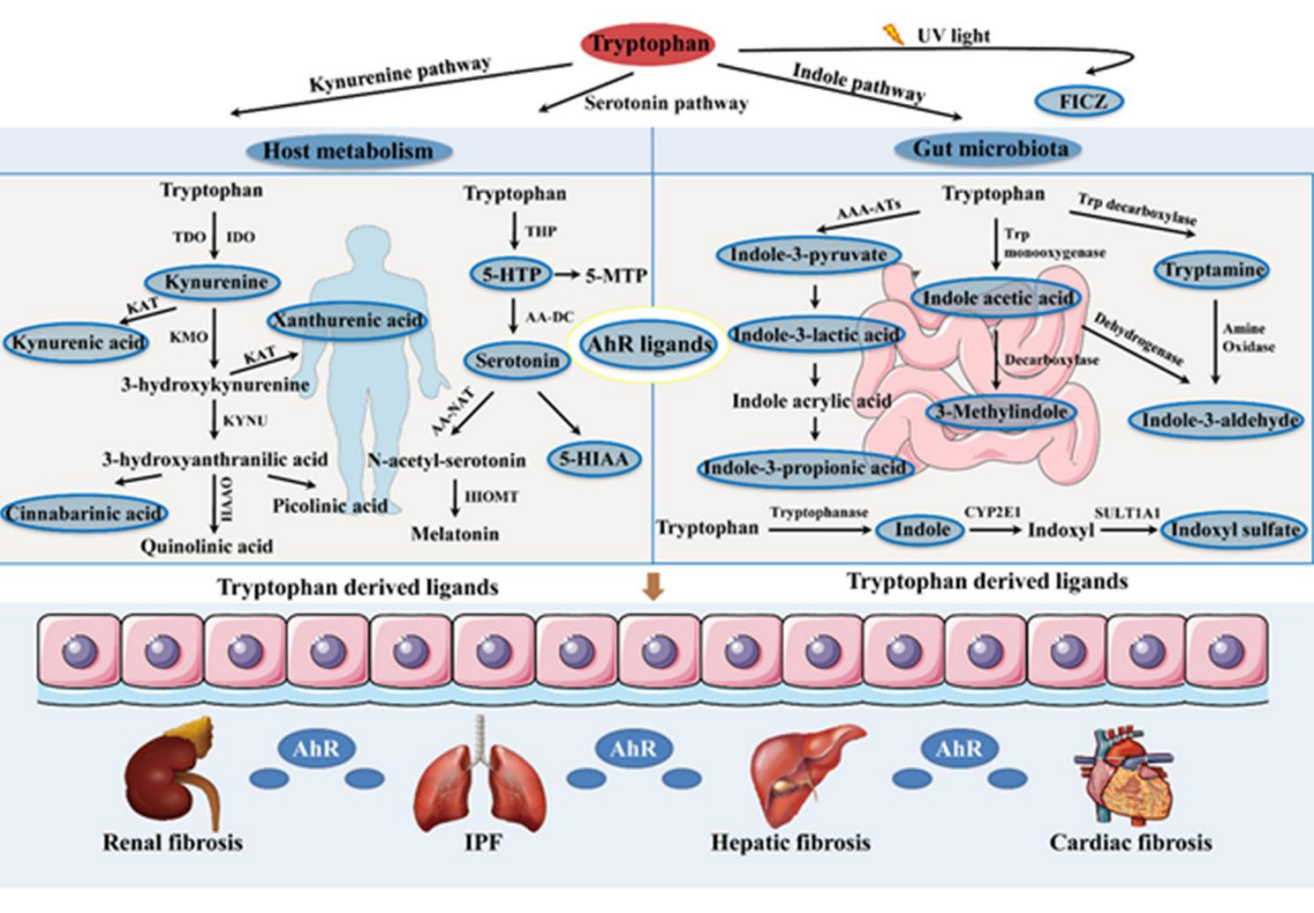
Schematic representation of the effect of tryptophan metabolism in age-related tissue fibrosis through AhR. Tryptophan is mainly metabolized through three pathways in the host and gut microbiota: the kynurenine pathway, serotonin pathway and indole pathway. The endogenous ligands for AhR that are surrounded in blue, including FICZ, kynurenine, kynurenic acid, xanthurenic acid, cinnabarinic acid, 5-HTP, serotonin, 5-HIAA, indole-3-pyruvate, indole-3-lactic acid, indole-3-propionic acid, indole acetic acid, 3-methylindole, tryptamine, indole-3-aldehyde, indole and indoxyl sulfate, may modulate fibrosis progression after binding with AhR. AAA-ATs: aromatic amino acid aminotransferases; AA-DC: aromatic amino acid decarboxylase; AA-NAT: arylalkylamine N-acetyltransferase; CYP2E1: cytochrome P450 2E1; FICZ: 6-formylindolo[3,2-b]carbazole; HAAO: 3-hydroxyanthranilic acid oxygenase; 5-HIAA: 5-hydroxy- indole-3-acetic acid; HIOMT: hydroxyindole O-methyltransferase; 5-HTP: 5-hydroxy-tryptophan; 5-MTP: 5-methoxy-tryptophan; IDO: indoleamine 2,3-dioxygenase; KAT: kynurenine aminotransferase; KMO: kynurenine 3-monooxygenase; KYNU: kynureninase; SULT1A1: sulfotransferase 1A1; TDO: tryptophan 2,3-dioxygenase; THP: tryptophan hydroxylase; Try: tryptophan.

## 2. Tryptophan metabolism: An overview

Tryptophan is an essential amino acid for humans that is acquired exclusively from dietary intake. Tryptophan and its metabolites have great effects on diverse physiological processes in which they serve as signaling molecules and building blocks of proteins to coordinate body responses to dietary and environmental cues [39-41]. Generally, tryptophan is metabolized via three metabolic pathways: the kynurenine pathway, serotonin pathway and indole pathway. Approximately 95% of tryptophan is converted to kynurenine, kynurenic acid, anthranilic acid, xanthurenic acid, cinnabarinic acid and quinolinic acid through the kynurenine pathway; 1-2% of tryptophan is degraded to 5-hydroxytryptophan, serotonin, 5-hydroxyindole-3-acetic acid and melatonin via the serotonin pathway; and 4-6% of tryptophan is metabolized to tryptamine, indole, indoxyl sulfate, indole-3-pyruvate, indole-3-lactic acid, indole propionic acid, indole acetic acid, 3-methylindole and indole-3-aldehyde through the indole pathway in the gut. Although AhR was first recognized as a receptor for exogenous aromatic hydrocarbons and hazardous environmental toxins, endogenous AhR ligands from tryptophan metabolism, including FICZ [42], kynurenine [43], kynurenic acid [44], xanthurenic acid [45], cinnabarinic acid [46], serotonin [47], 5-hydroxyindole-3-acetic acid [33], tryptamine, indole, indole propionic acid [48, 49], indole acetic acid, indoxyl sulfate [50], indole-3-aldehyde [9], indole-3-pyruvate [51], indole-3-lactic acid [52] and 3-methylindole [53], have also been identified (Fig. 1). In fact, the traditional AhR signaling pathway cannot fully explain all the biological processes attributed to AhR.

## 3. Role of tryptophan metabolism in age-related tissue fibrosis through AhR

### 3.1 Renal fibrosis

Chronic kidney disease (CKD) affects 8-16% of the population worldwide and is recognized as the 16th leading cause of death [54]. Aging leads to a significant decline in organ function, among which the kidney is particularly susceptible to age-associated damage [55]. Compared to young kidneys, aged kidneys have significantly increased fibrosis with impaired renal function, and it is highly recommended that kidney aging be explored along with the related mechanisms for the prevention of age-associated fibrosis [56]. The AhR signaling pathway has been reported to exacerbate aging phenotypes in different tissues, and here, we mainly focus on how tryptophan metabolism accelerates renal fibrosis via AhR [57, 58]. Epidemiological and experimental studies have indicated that the dysfunction of various metabolites is implicated in renal fibrosis, especially tryptophan-derived metabolites. Mesangial cells play important roles in the synthesis and degradation of type IV collagen, and serotonin can accelerate fibrosis by enhancing type IV collagen production in human mesangial cells, which is mediated by the activation of TGF-β/Smad signaling [59]. Moreover, AhR was activated in patients with CKD, and serum levels of kynurenic acid, kynurenine and quinolinic acid were increased with CKD severity [60]. Indoxyl sulfate is a representative protein-bound uraemic toxin, the excessive accumulation of which promotes CKD progression by activating both the canonical AhR and non-canonical AhR signaling pathways [61, 62], whereas indole-3-propionic acid could attenuate renal fibrosis by suppressing indoxyl sulfate-induced AhR expression [63]. Indole-3 acetic acid, another uremic toxin from the gut microbiota, was increased in parallel with CKD progression, while its level was substantially decreased and even normalized after kidney transplantation [64]. Furthermore, indole-3 acetic acid also contributed to the renal toxicity observed in CKD patients undergoing dialysis after binding to AhR, underscoring the importance of indole-3 acetic acid clearance in renoprotection [65].

Apart from the abovementioned factors, tryptophan metabolism is also highly associated with the development of complications in CKD, and a vicious cycle exists since CKD complications could exacerbate renal fibrosis. Cardiovascular disease is a global health threat, the risk of which is evidently increased in CKD [66]. Mounting evidence indicates that the progression of cardiovascular disease in CKD cannot be fully explained by traditional risk factors such as dyslipidemia, hypertension, and diabetic mellitus, and indoxyl sulfate-induced vascular inflammation may contribute to atherogenesis in CKD [67]. AhR-mediated tissue factor production [50] and immune dysfunction [68] also play pivotal roles in the pathogenesis of cardiovascular disease by accelerating atherosclerosis and provoking vascular endothelial damage, respectively. Similar results were observed for indole-3 acetic acid [50, 69]. Renal anemia is very common in CKD, and indoxyl sulfate could exacerbate anemia by inhibiting hypoxia-inducible factor-dependent erythropoietin production through the activation of AhR [70]. Additionally, iron deficiency was the major cause of anemia, and indoxyl sulfate also affected iron metabolism in CKD by participating in hepcidin regulation through AhR-dependent pathways, suggesting that indoxyl sulfate-induced AhR may be exploited as a therapeutic target for treating renal anemia [71]. Bone disorder is another CKD complication that appears at the early stage of renal failure, while the treatment for bone disorder remains a clinical challenge [72]. Kynurenine [73] and indoxyl sulfate [74] could exacerbate osteoporosis by decreasing bone strength and affecting osteoclastogenesis through AhR-associated mechanisms, respectively, the inhibition of which might provide new opportunities for the treatment of osteoporosis in CKD. Moreover, CKD patients with high indoxyl sulfate and kynurenine were predisposed to arteriovenous thrombosis after vascular injury through the uremic solute-AhR-tissue factor axis [75], hinting that AhR antagonists may also be novel targets for anti-thrombotic therapy.

### 3.2 Idiopathic pulmonary fibrosis

IPF is a fatal age-associated disease characterized by progressive and irreversible lung scarring, affecting approximately 130,000 people in the US and 3 million people worldwide, the prevalence and incidence of which dramatically increase with age [76]. In addition, the survival rate of IPF patients markedly decreases with age [77], and the median survival rate is less than 3 years [78, 79]. Although some risk factors have been identified, the etiology of IPF is still unknown, while age stands out as a major contributor to the pathogenesis since IPF is widely recognized as a disease of aging [80]. Therefore, it is critical to understand the age-associated mechanisms that play pivotal roles in disease pathogenesis with a growing population of elderly individuals. Kynurenine is increased with aging [81], and kynurenine elevation could signal through AhR to accelerate lung fibrosis by promoting epithelial-to-mesenchymal transition [82]. Reactive oxygen species (ROS) production and oxidative damage increase with age as well, which promotes the progression of age-related diseases, including IPF [83]. Zhang et al. discovered that AhR-deficient cells were susceptible to hypertoxic injury and that AhR activation could protect lung endothelial cells against ROS-induced damage [84]. However, Chen et al. uncovered that AhR downregulation ameliorated AhR-dependent oxidative damage caused by the environment and cigarette smoking exposure in lung epithelial cells [85], suggesting that lung endothelial cells respond differently to ROS than lung epithelial cells.

**Figure 2.**
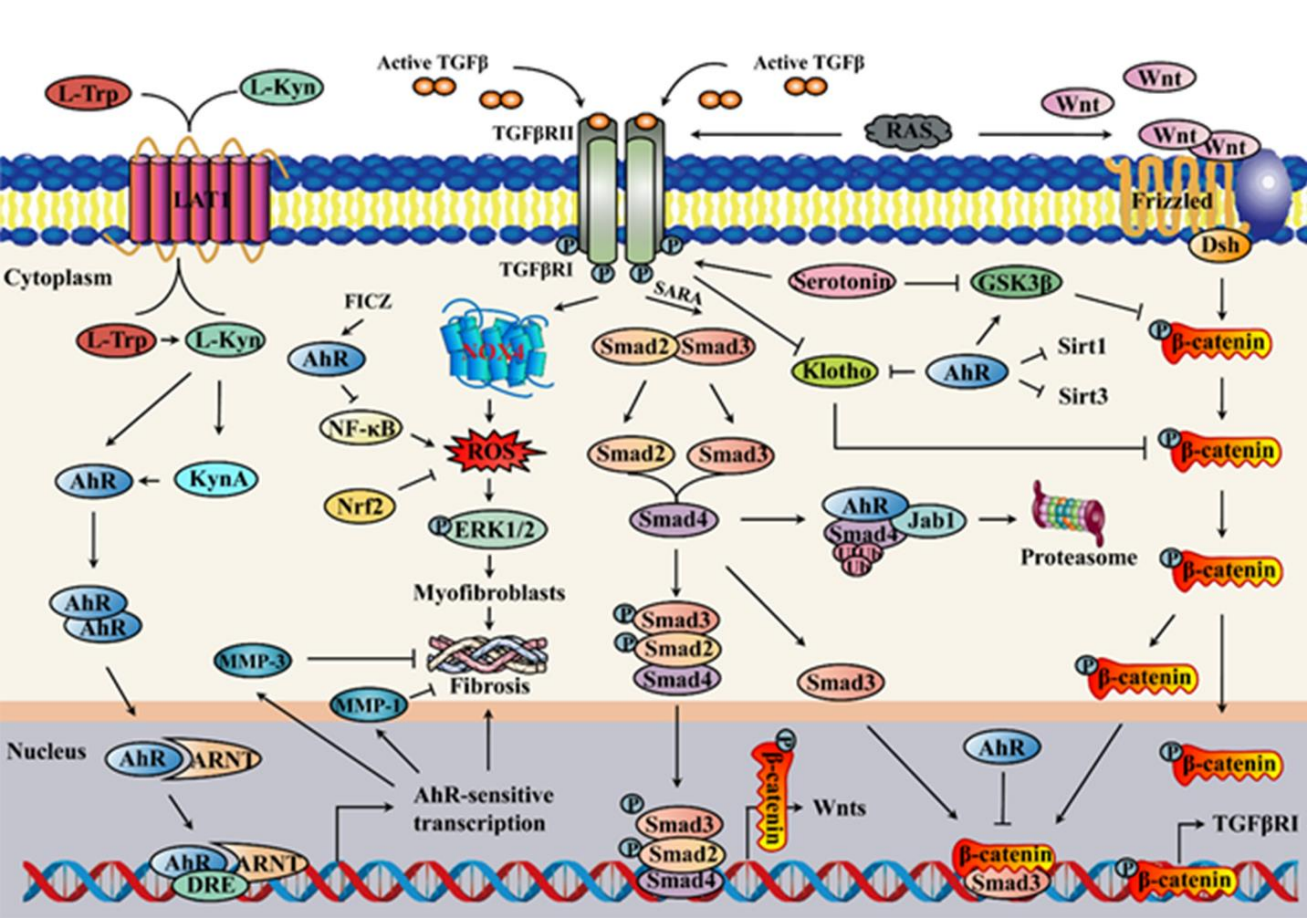
The crosstalk among AhR and other signalling pathways in tissue fibrosis. Both L-Kyn and KynA can activate AhR, and AhR is translocated to the nucleus upon interaction with a ligand, leading to AhR-sensitive transcription and fibrosis development. In addition to canonical AhR signalling, AhR also interacts with other pathways. TGF-β/Smad and Wnt/β-catenin signalling are other core pathways of fibrosis progression that play pivotal roles in renal fibrosis, idiopathic pulmonary fibrosis, hepatic fibrosis and cardiac fibrosis. AhR not only attenuates lung fibrosis by inducing Smad4 degradation in the proteasome but also alleviates hepatic fibrosis by disrupting the interaction of Smad3 and β-catenin. GSK3β is an inhibitor of β-catenin, and AhR may also inhibit lung fibrosis by maintaining GSK3β in an active form. Serotonin is a dedicated contributor to cardiac fibrosis and pulmonary fibrosis by activating the TGF-β signalling pathway. Moreover, it also enhances β-catenin signalling by inhibiting GSK3β. Inflammation also contributes greatly to fibrogenesis, and AhR activated by FICZ improves acute kidney injury by inhibiting NF-κB. Furthermore, AhR has been proven to suppress fibrosis by activating MMP-1/MMP-3 in fibroblasts, but whether it has a similar effect on ageing-related fibrosis remains to be determined. ARNT: aryl hydrocarbon receptor nuclear translocator; DRE: dioxin response element; ERK: extracellular signal-related kinase; Jab: Jun-activation domain binding protein; KynA: kynurenic acid; Kyn: kynurenine; LAT1: L-type amino acid transporter 1; L-Kyn: L-kynurenine; L-Trp: L-tryptophan; NOX: nicotinamide adenine dinucleotide phosphate oxidase; SARA: Smad anchor for receptor activation.

Inflammatory aging also contributes to the development of pulmonary disease in elderly individuals [86]. It is well known that regulatory T cells (Tregs) are critical for inflammation suppression [87], and AhR activation by FICZ attenuates IPF by increasing Tregs but inhibiting inflammatory T cell subsets in a bleomycin-induced lung fibrosis model [88]. Interestingly, pulmonary epithelial cells alleviated inflammation at least partially by suppressing their own IL-6 production through the kynurenine-AhR axis. Therefore, pulmonary epithelial cells may have a self-regulating system for inflammation [89]. Mesenchymal stem cells have been reported to protect against various diseases, and kynurenic acid pre-treatment enhances their therapeutic effect on lipopolysaccharide-induced acute lung damage through an AhR-associated mechanism [90]. 3-Methylindole is a pneumotoxicant derived from tryptophan metabolism in the human large intestine that causes acute pulmonary disease with highly selective toxicity to epithelial cells [91]. In addition to endogenous metabolism of tryptophan, cigarette smoke inhalation provides a considerable source of 3-methylindole exposure in humans, which directly brings the pneumotoxicant to its target organ. Weems et al. demonstrated that 3-methylindole led to DNA damage and induced cytochrome P450 enzyme expression in an AhR-dependent mechanism, providing mechanistic insights into 3-methylindole-mediated lung damage [92]. Of note, although AhR contributes to cigarette smoke- and environmental pollutant-induced lung damage, recent studies have indicated that AhR negatively regulates the progression of lung cancer. Lee et al. discovered that AhR overexpression attenuated lung cancer invasion by inducing Smad4 ubiquitination and proteasome degradation after binding to Smad4 under non-ligand conditions in lung cancer cells, providing new insight into the role of AhR in cancer formation and a novel target for therapeutic intervention [93] (Fig. 2). Individuals who suffering from SARS-CoV-2 infections are also at high risk of organ injure processing including the lung, heart, kidney. One case from clinical experience reported that idiopathic pulmonary fibrosis post-COVID-19 infection was the exacerbating factor causing progression of disease. Even she was treated with reversible inflammation, unfortunately, there was no improvement clinically [35]. It's because of CoV persistently activate AhRs, this may lead to up-regulation of multiple sets of downstream effectors resulting in pulmonary fibrosis. During late phase of the IPF, cytokine elevation related to persistent activation of AhRs by CoV, can also induce tissue injury in diverse organs, and contribute to worsening of clinical manifestations and disease outcomes [38].

### 3.3 Hepatic fibrosis

Hepatic disease is the fifth leading cause of premature life lost in the Western world, with irreversible damage caused by liver fibrosis, and ultimately cirrhosis, a primary contributor to mortality [94]. Aging is intimately associated with the physiological decline of the liver, which involves changes in hepatic structure and hepatic function, thus leading to the development of age-related hepatic diseases [95]. AhR is highly expressed in the liver, while the role of AhR in hepatic fibrosis is controversial since both loss and gain of AhR expression could lead to hepatic fibrosis. On the one hand, AhR has a great influence on liver development, and mice lacking AhR showed spontaneous hepatic fibrosis with significant liver architecture alteration, suggesting that the basal AhR activity may suppress fibrotic phenotypes in vivo [96]. On the other hand, AhR activation sensitized mice to hepatic fibrosis by regulating profibrotic pathways [97, 98]. Considering that there are multiple cell types in the liver, including hepatocytes, hepatic stellate cells and Kupffer cells, it is reasonable to postulate that AhR may have a cell type-specific role in hepatic fibrosis. Activated hepatic stellate cells are dominant contributors to hepatic fibrosis since they are major cells responsible for the production of extracellular matrix proteins and profibrogenic cytokines [99]. Yan et al. discovered that AhR knockout in hepatic stellate cells was sufficient to cause spontaneous hepatic fibrosis, whereas treatment of mice with non-toxic AhR ligand ameliorated hepatic fibrosis by preventing the interaction of Smad3 and β-catenin [100] (Fig. 2). Notably, similar results were not observed in hepatocytes/Kupffer cells [100], and the function of AhR can be differentially altered by binding to different ligands, which may explain the contradictory role of AhR in hepatic fibrosis.

Kynurenic acid exerted a hepatoprotective effect on thioacetamide-induced liver failure partly by mediating AhR, and the use of a diet containing high kynurenic acid can be beneficial to the recovery of liver injury, as kynurenic acid is a constituent of foods and is found in considerable amounts in selected products [101]. Indole-3-acetate may ameliorate hepatic fibrosis by inhibiting the release of pro-inflammatory cytokines through an AhR-associated mechanism [102]. Chronic alcohol consumption is an important contributor to liver-related deaths [103], while alcohol-induced hepatic fibrosis was reversed by AhR agonists generated by tryptophan metabolism in the intestine [32]. Given that there is no treatment that reverses alcohol-induced liver lesions except for liver transplantation, the modulation of AhR signaling by supplementation with AhR ligand-producing bacteria may hold promise in inspiring new therapeutic therapies for alcoholic liver disease. Additionally, emerging studies demonstrated that stanniocalcin 2 was a novel AhR target gene specifically responsive to cinnabarinic acid, and AhR-dependent stanniocalcin 2 activations induced by cinnabarinic acid protected against chemical insult or oxidative stress-induced liver damage [104, 105]. 3-Methylindole is a pulmonary toxin that activates AhR, while it acts as an antagonist of TCDD-induced AhR activation, suggesting that 3-methylindole is a partial AhR agonist and potentially influences liver function alone or in combination with other AhR ligands [106]. Approximately 90% of kynurenine is generated by indoleamine 2,3-dioxygenase (IDO), and the level of kynurenine is increased in the liver after CCl_4_ treatment accompanied by the activation of AhR signaling, whereas IDO2 inhibition attenuates hepatic fibrosis, hinting that kynurenine produced by IDO2 exacerbates liver injury by activating AhR [107]. Astonishingly, kynurenine could restrain fibrosis by upregulating MMP-1/MMP-3 and further promoting excessive fibrotic collagen degradation through an AhR-dependent mechanism, highlighting the potentially significant therapeutic value of kynurenine intervention for fibrotic disorders [16, 108, 109] (Fig. 2). However, it is unknown whether kynurenine has a similar effect in the liver, and future studies are urgently needed.

### 3.4 Cardiac fibrosis

Cardiac fibrosis is a hallmark of myocardial remodeling and is deeply implicated in the development of heart failure [110]. Aging induces cardiac structural changes linked to increased extracellular matrix protein deposition, leading to progressive interstitial fibrosis [111]. Gut microbiota dysfunction induced by excessive cardiac pressure contributes to adverse myocardial remodeling, while tryptophan metabolites ameliorate cardiac fibrosis in heart failure by activating AhR signalling [112]. In addition, AhR was cardioprotective against doxorubicin-induced cardiotoxicity [113], while AhR ablation promoted angiotensin II-induced cardiac fibrosis [114]. Kynurenine is a uremic toxin that plays a vital role in cardiac hypertrophy by mediating AhR, and the production of cardiac hypertrophy markers is reversed by AhR knockdown [115], suggesting that kynurenine and AhR are important mediators of cardiac injury that may be pharmacologically manipulated in vivo. In addition to the canonical AhR signaling pathway, kynurenine also accelerates cardiac fibrosis through the non-canonical AhR signaling pathway. Lekawanvijit et al. discovered for the first time that indoxyl sulfate had pro-fibrotic, pro-inflammatory and pro-hypertrophic effects, indicating that indoxyl sulfate might contribute to adverse cardiac remodeling by modulating cardiac fibroblasts and NF-κB signaling [116].

### 3.5 The crosstalk among AhR and other signaling pathways in aging-related fibrosis

TGF-β/Smad signaling is widely recognized as the core pathway of fibrosis, and AhR may attenuate lung fibrosis by inducing Smad4 degradation in the proteasome under non-ligand conditions [93]. Wnt/β-catenin signaling is another prominent signaling pathway that affects tissue fibrosis with aging, and Wnt/β-catenin dysfunction also plays a paramount role in aging-related tissue fibrosis [3]. Yan et al. discovered that Smad3 could promote fibrogenesis by binding to β-catenin, while AhR alleviated hepatic fibrosis by disrupting the interaction of Smad3 and β-catenin [100]. GSK3β is an inhibitor of β-catenin that induces β-catenin degradation by forming a complex together with axin and *adenomatosis polyposis coli* [117]. AhR could maintain GSK3β in an active form, thus suppressing epithelial-mesenchymal transition in lung cancer cells, which may be used to alleviate lung fibrosis [118]. Serotonin not only promoted cardiac fibrosis and pulmonary fibrosis by activating the TGF-β signaling pathway [119, 120] but also enhanced β-catenin signaling by inhibiting GSK3β [121]. In addition, klotho is an aging suppressor that represses Wnt/β-catenin signaling by binding to Wnt ligands, whereas klotho expression is markedly inhibited by AhR and TGF-β/Smad signaling [122]. Sirtl and Sirt3 are other aging suppressors, the loss of which contributes much to fibrosis progression [123, 124], and AhR could also signal with Sirtl [29] and Sirt3 [30]. Moreover, inflammation also plays an important role in fibrosis progression, and AhR signaling activated by FICZ can improve acute kidney injury by inhibiting the NF-κB pathway [125]. Furthermore, AhR has been reported to promote fibrillar collagen degradation by activating MMP1/MMP3 in fibroblasts, but whether it has a similar effect on renal fibrosis, idiopathic pulmonary fibrosis, hepatic fibrosis and cardiac fibrosis remains to be determined. In brief, AhR signaling is a dedicated modulator of aging-related diseases, and it also interacts with other signaling pathways, the intervention of which may yield promising candidates for anti-fibrotic therapies (Fig. 2).

## 4. Ongoing clinical trials for age-related tissue fibrosis

### 4.1 The trials for renal fibrosis

Worldwide estimates indicate that approximately 700 million individuals suffer from CKD, which contributes greatly to diminished quality of life and shorter life expectancy [126]. Despite the widespread availability of laboratory tests to identify patients with abnormal kidney function, very few clinical trials have been conducted to explore kidney diseases [127]. Until recently, angiotensin-converting-enzyme inhibitors and angiotensin-receptor blockers were the only classes of medicine that have been effective in slowing kidney function decline [128-131]. Empagliflozin [132], canagliflozin [133] and sotagliflozin [134] have been reported to reduce the risk of adverse events in patients with CKD and diabetes by inhibiting sodium-glucose cotransporter 2 (SGLT2), supporting the potential use of sodium-glucose cotransporter 2 inhibitors combined with other drugs in the treatment of CKD and diabetes. Similar results were observed in atrasentan [135] and finerenone [136]. It seems that the benefits of SGLT2 inhibitors are independent of the glucose-lowering effects, and their renal protective effects may persist in CKD patients caused by factors other than type 2 diabetes [137, 138]. Heerspink et al. investigated the effect of dapagliflozin (10 mg once a day) in CKD patients with or without type 2 diabetes and demonstrated that dapagliflozin significantly improved the clinical outcomes of CKD patients by inhibiting SGLT2 regardless of the absence or presence of diabetes [139].

In addition, higher plasma marine n-3 fatty acids were inversely associated with renal fibrosis development and independently associated with better survival in 1990 Norwegian renal transplant recipients, suggesting that marine n-3 fatty acid supplementation may assist renal function recovery [140]. However, meta-analyses of randomized controlled trials found that there was no statistically significant effect of marine n-3 fatty acids on renal function [141, 142]. Considering that the efficacy of marine n-3 fatty acids may be hampered by low sample sizes, Eide et al. conducted the largest randomized clinical trial to investigate the effect of marine n-3 fatty acids (2.6 g daily for 44 weeks) on renal function during the first year after kidney transplantation [143]. Astonishingly, marine n-3 fatty acid supplementation was safe, while it did not improve renal function compared to the controls. Collectively, the currently available randomized clinical trials cannot provide sufficient evidence to recommend marine n-3 fatty acid therapy to treat kidney disease. Given that the safety of marine n-3 fatty acids is well documented and its beneficial effect on cardiac fibrosis [144], more studies with longer durations are required to determine whether marine n-3 fatty acids could preserve renal function. Moreover, contrast-induced nephropathy is a serious health problem with increased morbidity and mortality, especially in patients with renal insufficiency. Sarpogrelate is a selective serotonin receptor subtype 2A antagonist that can block serotonin-induced platelet aggregation and has an anti-inflammatory effect, which has been shown to reduce renal fibrosis in animal models [145, 146], while the clinical outcome of sarpogrelate in contrast induced nephropathy is disappointing [147]. Of most patients who experienced an adverse event during the above trials, fracture and gastrointestinal discomfort were reported by more patients in the treat group. The severe adverse events and conflications are presented in Table1.

**Table 1:** The ongoing clinical trials for age-related tissue fibrosis

Drug	Target	Phase	Trial	Result	Ref.	Time	ADR	Complication
Renal fibrosis								
Empagliflozin	SGLT2	-	NCT01131676	Positive	[132]	2.6y△	Genital infection, volume depletion, bone fracture	-
Canagliflozin	SGLT2	-	NCT02065791	Positive	[133]	2.5y	Amputation, fracture, diabetic ketoacidosis	-
Sotagliflozin	SGLT2	-	NCT03315143	Positive	[134]	14.2m△	Diarrhea, genital mycotic infections, volume depletion	-
Atrasentan	Endothelin A receptor	Phase 3	NCT01858532	Positive	[135]	36m	Fluid retention, anaemia, cardiac toxicity	-
Finerenone	MR	Phase 3	NCT02540993	Positive	[136]	36m	Hyperkalemia, modest reduction in blood pressure, change in bodyweight	Hyperkalemia, irrespective of history of CVD
Dapagliflozin	SGLT2	-	NCT03036150	Positive	[139]	30m	Renal-related adverse event, volume depletion, fracture	-
Marine n-3 FAs	-	-	NCT01744067	NSB	[143]	44w	Gastrointestinal discomfort, acute rejection episode, post transplantation diabetes mellitus	-
Sarpogrelate*	5-HT2A	-	NCT01165567	NSB	[147]	4w	-	-
Idiopathic pulmonary fibrosis								
Pirfenidone plus nintedanib	TKs	Phase 4	NCT02598193	Positive	[151]	24w	Nausea, diarrhoea, vomiting	-
Pirfenidone plus nintedanib	TKs	-	NCT02579603	Positive	[152]	12w	Nausea, diarrhea, vomiting	-
PBI-4050	GPR	Phase 2	NCT02538536	Positive	[154]	12w	Diarrhoea, nausea, headache	-
GSK2126458	PI3K/mTOR	-	NCT01725139	Positive	[155]	5d		
GSK2126458	PI3K/mTOR	-	2012-001376-11	Positive	[156]	8d	Diarrhoea, nausea, hyperglycaemia	-
Thalidomide	-	-	NCT00600028	Positive	[157]	24w	Constipation, dizziness, malaise	Constipation, bradycardia
Omeprazole	Proton pump	-	NCT02085018	Positive	[158]	86d	Lower respiratory tract infection, abdominal pain, cough	-
Mesenchymal stromal cells	-	Phase 1b	NCT01385644	Positive	[159]	6m	Lightheadness,flushed in face, blurred vision	Worsing lung function, oxygenation
Pamrevlumab	CTGF	Phase 2	NCT01890265	Positive	[160]	48w	Respiratory tract infection, cough, dyspnoea	Respiratory related
GLPG1690	Autotaxin	Phase 2a	NCT02738801	Positive	[161]	12w	Lower respiratory tract infection, nasopharyngitis, cough	-
BMS-986020	LPA1	Phase 2	NCT01766817	Positive	[162]	26w	Cough, headache, fatigue	IPF related
Pentraxin 2	Monocyte	Phase 2	NCT02550873	Positive	[163]	28w	Cough, fatigue, nasopharyngitis	Respiratory decline
FG-3019	CTGF	Phase 2	NCT01262001	Positive	[164]	45w	Cough, fatigue, dyspnoea	IPF, pneumonia
BIBF 1120	TKs	Phase 2	NCT00514683	Positive	[165]	52w	Diarrhea, cough, nausea	-
Acetylcysteine	-	-	20130109-2	Positive	[184]	26w	Cough, drug-induced pneumonitis	-
Bosentan	-	-	NCT00391443	NSB	[166]	17.9w	Cough, dyspnea, bronchitis	Respiratory failure
Interferon γ-1b	-	-	NCT00075998	NSB	[167]	577d	Cough, headache, influenza-like illness	Respiratory-related, sepsis or multiorgan failure , cardiac
SAR156597	Interleukin	Phase 2b	NCT02345070	NSB	[168]	52w	IPF, cough, diarrhoea	IPF, pneumonia, septic shock
Tralokinumab	Interleukin-13	Phase 2	NCT01629667	NSB	[169]	64w	Cough, IPF, upper respiratory tract infection	IPF, Pneumonia, cardiac arrest
Carbon monoxide	-	Phase 2a	NCT01214187	NSB	[170]	12w	Respiratory/thoracic/mediastinal, nervous system, gastrointestinal	-
Simtuzumab	LOXL2	Phase 2	NCT01769196	NSB	[171]	82w	IPF, fatigue, atrial fibrillation	IPF exacerbation or progression
Carlumab	CCL2	Phase 2	NCT00786201	NSB	[172]	48w	Respiratory, thoracic and mediastinal disorders, Infections and infestations, Cardiac disorders	-
Macitentan	Endothelin receptor	Phase 2	NCT00903331	NSB	[173]	12m	IPF, dyspnoea, cough	IPF worsening, respiratory failure
Imatinib	TKs	Phase 2	NCT00131274	NSB	[174]	96w	Periorbital edema, diarrhea/cramping, nausea	Acute worsening of IPF
Thrombomodulin α	-	Phase 3	NCT02739165	NSB	[175]	14d	Insomnia, delirium, hyperglycemia	The progression or recurrence of AE-IPF
Sildenafil	PDE5	-	NCT00517933	NSB	[176]	24w	Worsening of idiopathic pulmo nary fibrosis, worsening of dyspnea, pneumonia	-
Sildenafil plus nintedanib	PDE5; TKs	-	NCT02802345	NSB	[177, 178]	24w, 24w	Diarrhea, decreased appetite, nausea	-, cardiovascular event
Sildenafil plus pirfenidone	PDE5	Phase 2b	NCT02951429	NSB	[179]	52w	Gastrointestinal disorder, clinically significant vascular event, hypotension event	-
Lebrikizumab	Interleukin-13	Phase 2	NCT01872689	NSB	[180]	52w	Cough, IPF, dyspnoea	IPF, pulmonary embolism
Lebrikizumab plus pirfenidone	Interleukin-13	Phase 2	NCT01872689	NSB	[180]	52w	Nasopharyngitis, upper respiratory tract infection, cough	IPF, pneumonia, hot disease
Acetylcysteine	-	-	NCT00650091	NSB	[183]	60w	Respiratory, infectious, cardiac	Respiratory
Ambrisentan	Endothelin A receptor	Phase 3	NCT00768300	Negative	[181]	34.7w	Edema peripheral, upper respiratory tract infection, headache	IPF, dyspnea, pneumonia
Warfarin	-	-	NCT00957242	Negative	[182]	48w	Bleeding	Cardiovascular, pulmonary hypertension, pneumonia
Acetylcysteine plus pirfenidone	TGF-β1	Phase 3	UMIN000015508	Negative	[185]	48w	Pneumonia, photosensitivity, decreased appetite	-
Acetylcysteine plus pirfenidone	-	Phase 2	2012-000564-14	Negative	[186]	24w	Photosensitivity, cardiac, diarrhoea	worsening IPF
Hepatic fibrosis								
Elafibranor	PPAR	Phase 2	NCT01694849	Positive	[188]	52w	Nausea, heahache, diarrhea	serum creatinine
GS-0976	ACC	Phase 2	NCT02856555	Positive	[189]	12w	Nausea, heahache, diarrhea	night sweats, hypertriglyceridemia
Resveratrol	-	-	NCT02030977	Positive	[190]	12w	-	-
GR-MD-02	Galectin 3	Phase 1	-	Positive	[192]	6/7w	Headache, dizziness, diarrhoea	-
Diacerein	Interleukin-1	-	NCT02242149	Positive	[196]	24m	-	-
Dapagliflozin	SGLT2	-	UMIN000022155	Positive	[197]	24w	-	-
Candesartan	Angiotensin	-	NCT00990639	Positive	[198]	6m	-	-
Capsule oxymatrine	-	-	-	Positive	[199]	52w	Nausea, rash, chest discomfort	-
Interferon-γ	TGF-β1	-	-	Positive	[200]	6m	Limb pain, nausea, muscle pain	-
Silibinin	-	Phase 2	NCT01518933	Positive	[204]	14d	Feeiling hot, diarrhea, headache	-
Telbivudine	-	-	NCT00877149	Positive	[206]	261w	-	-
Synbiotic agents	-	Phase 2	NCT01680640	NSB	[191]	10-14m	Weight loss	-
GR-MD-02	Galectin 3	Phase 2	NCT02462967	NSB	[193]	52w	Nausea, diarrhea, nasopharyngitis	Spasmodic cough#
GR-MD-02	Galectin 3	Phase 2	NCT02421094	NSB	[194]	16w	-	-
Peg-interferon α-2a	-	-	NCT00122616	NSB	[201]	96w	Liver failure with ascites and edema, neutropenia, anemia	-
Oltipraz	TGF-β1	Phase 2	NCT00956098	NSB	[202]	24w	-	-
Simtuzumab	LOXL2	Phase 2	NCT01707472	NSB	[203]	22w	Fever, headache, asymptomatic elevations in amylase and lipase	-
Cardiac fibrosis								
Empagliflozin	SGLT2	-	NCT03057977	Positive	[208]	124w	Uncomplicated genital tract infection, hypoglycemia, lower limb amputation	-
Dapagliflozin	SGLT2	-	NCT03036124	Positive	[209]	8w	Volume depletion, renal AE, fracture	-
Omega-3 PUFAs	-	-	-	Positive	[210]	8w	-	-
Omega-3 acid ethyl ester	-	-	NCT00729430	Positive	[144]	6m	Nausea, fishy taste, bleeding	-
Lisinopril	ACE	-	1262GR/0006	Positive	[214]	24w	-	-
Torasemide	Type I collagen	-	NCT00409942	NSB	[216]	32w	Diabetes mellitus, hyperuricemia, hypokalemia	-

* Drugs that interfere with the tryptophan-AhR pathway,

△ Time that the median duration if treatment,

# Complication probably related to study drug ACC: acetyl-coenzyme A carboxylase; ACE: angiotensin-converting enzyme; CCL2: CC-chemokine ligand 2; CTGF: connective tissue growth factor; GPR: G-protein coupled receptor; 5-HT2A: serotonin receptor subtype 2A; LOXL2: lysyl oxidase-like 2; LPA1: lysophosphatidic acid receptor 1; MR: mineralocorticoid receptor; mTOR: mammalian target of rapamycin; NSB: no significant benefit; PDE: phosphodiesterase; PI3K: pan-PI3 kinase; PPAR: peroxisome proliferator-activated receptor; PUFAs: polyunsaturated fatty acids; ROS: reactive oxygen species; TGF-β: transforming growth factor-β; TKs: tyrosine kinases

### 4.2 The trials for idiopathic pulmonary fibrosis

The development of pirfenidone and nintedanib has been an exciting landmark in the treatment of IPF, and both of them are recommended for clinical use in the current clinical practice guidelines [148]. Unfortunately, although pirfenidone and nintedanib slow IPF progression, the disease continues to progress, indicating that additional therapies are needed to cure this deadly disease. Current efforts are primarily focused on developing new drugs and the combination of new agents with available therapies. Pirfenidone and nintedanib are thought to mediate different aspects of fibrotic cascades, the combination of which might provide additive effects on IPF outcomes compared with either monotherapy [149, 150]. Flaherty et al. assessed the safety of treatment with pirfenidone and nintedanib in 89 patients and discovered that treatment with pirfenidone (1602-2403 mg/day) and nintedanib (200-300 mg/day) for 24 weeks was well tolerated by the majority of IPF patients, supporting further research into combination regimens for patients with IPF [151]. Similar results were observed in another open-label, randomized trial [152]. PBI-4050 is a novel small-molecule compound that demonstrates anti-fibrotic effects in several fibrosis models, including lung fibrosis [153]. Khalil et al. conducted the first clinical study of PBI-4050 in patients with IPF and found that no safety concerns arose among patients receiving PBI-4050 alone or in conjunction with pirfenidone/nintedanib [154]. However, there was a drug-drug interaction between pirfenidone and PBI-4050, while the pharmacokinetic profiles were similar among patients with PBI-4050 alone or in conjunction with nintedanib, encouraging further study of PBI-4050 alone or PBI-4050 plus nintedanib in patients with IPF [154]. Phosphatidylinositol 3-kinase signaling, and mammalian target of rapamycin also play vital roles in IPF pathogenesis, the inhibition of which has been shown to attenuate fibrosis progression in randomized and placebo-controlled trials [155, 156]. Persistent, non-productive and often disabling cough that has no effective treatment is one of the most prominent symptoms of IPF, affecting up to 80% of IPF patients [157]. Thalidomide [157] and omeprazole [158] were reported to alleviate cough and improve the respiratory quality of patients with IPF, while large-scale prospective clinical trials are still warranted to further evaluate their efficacy and safety.

Moreover, mesenchymal stromal cells [159], pamrevlumab [160], GLPG1690 [161], BMS-986020 [162], pentraxin 2 [163], FG-3019 [164] and BIBF 1120 [165] also showed potential clinical benefits for patients with IPF in phase 1/2 trials. However, although a series of anti-fibrotic agents exhibited a promising future in clinical use, many ongoing clinical trials failed to demonstrate benefit in treating IPF patients compared to placebo, such as bosentan [166], interferon γ-1b [167], SAR156597 [168], tralokinumab [169], inhaled carbon monoxide [170], simtuzumab [171], carlumab [172], macitentan [173], imatinib [174] and thrombomodulin α [175]. Additionally, both sildenafil monotherapy [176] and in combination with nintedanib [177, 178] or pirfenidone [179] did not provide a significant benefit in patients with IPF. Similar results were observed for lebrikizumab [180]. Of note, endothelin-1 and the coagulation cascade were believed to be implicated in the pathogenesis of IPF, while treatments with ambrisentan [181] and warfarin [182] were associated with even worse outcomes in IPF patients and should not be used for clinical use. Furthermore, up to one-third of IPF patients in Europe are reported to treat IPF by using pirfenidone and acetylcysteine, while the efficacy of acetylcysteine on IPF remains controversial [183, 184], and the combination of acetylcysteine with pirfenidone may be harmful to patients with IPF [185, 186]. Therefore, more large-scale clinical trials are urgently needed to further evaluate its safety and efficacy. The most common reported ADRs were nausea, diarrhea, and cough ff most patients who experienced an adverse event during the above trials. The severe adverse events and conflictions are presented in Table1.

### 4.3 The trials for hepatic fibrosis

Non-alcoholic fatty liver disease is the most common liver disease with complex pathophysiology that is characterized by hepatic steatosis and can potentially progress to advanced fibrosis or end-stage liver disease, affecting approximately 24% of the population worldwide [187]. Elafibranor [188], GS-0976 [189] and resveratrol [190] were proven to be effective in hepatic fibrosis regression for patients with non-alcoholic fatty liver disease, while patients receiving a synbiotic combination (prebiotic and probiotic) failed to slow hepatic fibrosis progression [191]. Galectin 3 contributed greatly to the pathogenesis of hepatic fibrosis, including inflammation and fibrosis caused by non-alcoholic fatty liver disease, whereas GR-MD-02 alleviated liver fibrosis by inhibiting galectin 3 and was well tolerated in phase I studies [192]. Unfortunately, the results of two-phase II studies evaluating GR-MD-02 in hepatic fibrosis were disappointing, as it did not exhibit robust efficacy in fibrosis improvement, which needs to be validated in future studies [193, 194]. In addition, type 2 diabetes and non-alcoholic fatty liver disease, both of which severely threaten human health, share many physiopathological pathways [195]. Diacerein [196] and dapagliflozin [197] have been reported to reduce hepatic fibrosis in diabetic patients with non-alcoholic fatty liver disease in randomized controlled trials, fueling considerable enthusiasm for the conduction of further clinical trials. Moreover, alcohol is a primary cause of hepatic fibrosis, and chronic alcohol consumption contributes much to the development of end-stage liver disease. The administration of candesartan plus ursodeoxycholic acid significantly improved hepatic fibrosis in patients with alcoholic liver disease without serious side effects, suggesting that candesartan can be beneficial to alcohol-induced hepatic fibrosis [198]. Hepatic fibrosis due to chronic viral infection also has an enormous socioeconomic impact. In addition to strategies targeting virus elimination, inhibition, or prevention of fibrogenesis is amenable. Oxymatrine capsule [199] and interferon-γ [200] were effective in the treatment of hepatic fibrosis caused by chronic viral hepatitis, while no significant changes were observed in hepatic fibrosis in patients receiving peg-interferon α-2a [201], oltipraz [202] and simtuzumab [203]. Rendina et al. discovered that silibinin monotherapy had a significant anti-viral effect in patients with established hepatitis C virus recurrence, and there was no interaction with other drugs for the first time, encouraging the evaluation of silibinin in combined therapy with other anti-viral drugs [204]. Furthermore, although anti-viral therapy is important for ameliorating liver fibrosis in patients with chronic viral infection, the complete regression of hepatic cirrhosis remains an intractable problem, and many patients with hepatitis B infection will progress to cirrhosis within 5 years [205]. Hou et al. demonstrated that prolonged telbivudine treatment led to durable virologic inhibition and significant improvement in hepatic fibrosis, thus achieving the long-term goals of anti-viral treatment in patients with chronic hepatitis B infection, which may help resolve the problem of cirrhosis progression in hepatitis B-infected persons [206]. During the above clinical trials, side effects mostly occurred with nausea, diarrhea, headache and so on during the treatment (Table 1).

### 4.4 The trials for cardiac fibrosis

Heart failure is a major public health problem with substantial morbidity and mortality, the burden of which has increased to 23 million people worldwide [207]. Empagliflozin [208] and dapagliflozin [209] have been reported to reduce the risk of cardiovascular death and hospitalization for heart failure by inhibiting SGLT2 in randomized, double-blind, controlled trials. Omega-3 polyunsaturated fatty acids have multiple cardioprotective effects, and the beneficial role of omega-3 poly-unsaturated fatty acids in cardiac fibrosis in ischemic heart failure patients has recently been demonstrated in a double-blind, placebo-controlled trial [210]. Additionally, Bobak et al. investigated the effect of omega-3 acid ethyl esters on patients with acute myocardial infarction and found that treatment with high-dose omega-3 acid ethyl esters could ameliorate myocardial fibrosis in addition to the current guideline-based standard of care [144]. Moreover, a prolonged state of ventricular pressure overload, caused by hypertension or aortic valve diseases, promotes myocardial remodeling that progresses to cardiac fibrosis and heart failure [211]. Therefore, suppressing hypertensive ventricular remodeling may assist cardiac fibrosis recovery. Previous studies uncovered that lisinopril could restrain cardiac fibrosis by halting angiotensin-converting enzyme in spontaneously hypertensive rats, but whether cardiac fibrosis can also be alleviated in patients with hypertensive heart disease was unknown [212, 213]. Brilla et al. first evaluated the effect of lisinopril on patients with hypertensive heart disease and demonstrated its efficacy in cardiac fibrosis regression, providing evidence for large-scale clinical trials [214]. However, to date, no further trials have been conducted. Furthermore, torasemide was reported to alleviate cardiac fibrosis in biopsy specimens of hypertensive patients with symptomatic heart failure [215], while the results of a randomized, open-label trial evaluating prolonged-release torasemide for cardiac fibrosis of hypertensive patients with chronic heart failure were disappointing [216]. Considering the differential effect of dietary and other treatments on the efficacy variables of open-label trials, more clinical trials are warranted to further evaluate torasemide’s efficacy. The adverse drug reactions mainly included hypoglycemia, bleeding, nausea etc in above trials. The main events ae listed in Table1.

## 5. Concluding remarks

Fibrosis is a common sequela of organ injury that results in organ dysfunction and potential death. Although it accounts for a considerable burden of diseases globally, there are no effective therapeutic strategies that successfully mitigate fibrosis. Aging-related tissue fibrosis, including renal fibrosis, IPF, hepatic fibrosis and cardiac fibrosis, has become more acute with the aging of populations. Moreover, COVID-19 is spreading rapidly across the world with substantial morbidity and mortality, which has given rise to a global pandemic of unprecedented proportions in the modern era since it is highly contagious and has an enormous impact on human health [217-219]. As the spread of SARS-CoV-2 is increasingly becoming out of control, the epidemic is now in the most formidable phase. COVID-19 is characterized by multiorgan failure, and patients with renal fibrosis, IPF, hepatic fibrosis and cardiac fibrosis are susceptible to COVID-19 and to severe outcomes from the disease [220-224]. Therefore, it is essential to develop mechanism-based therapies that effectively halt fibrosis progression, especially in the background of the COVID-19 outbreak.

Accumulating evidence indicates that the gut microbiota is an important contributor to the metabolic health of humans, the dysregulation of which has a pathogenic effect on multiple diseases, including tissue fibrosis [225-228]. AhR signaling also plays a crucial role in modulating fibrosis progression [25, 26]. Many metabolites are involved in the crosstalk between the host and gut microbiota, among which tryptophan metabolites are dedicated players in fibrogenesis by acting as endogenous AhR ligands. However, to date, only a few studies have shed light on the impact of tryptophan metabolites on fibrosis modulation through AhR, especially for renal fibrosis and cardiac fibrosis. In addition, natural products are fertile ground for drug discovery, while only a few studies have evaluated the effect of natural products on fibrosis regression by AhR-associated mechanisms over the past few decades [229-231]. Randomized trials that provide evidence-based guidelines for clinical decisions are prerequisites before reaching clinical application. Unfortunately, there are fewer clinical trials for renal fibrosis, hepatic fibrosis, and cardiac fibrosis than for IPF, and more than half of the trials for IPF have failed to demonstrate benefit in IPF patients. Given the enormous influence of AhR signaling in fibrosis development and the limited number/efficacy of ongoing clinical trials for aging-related tissue fibrosis, it is highly recommended to exploit novel therapies targeting the AhR signaling pathway.
